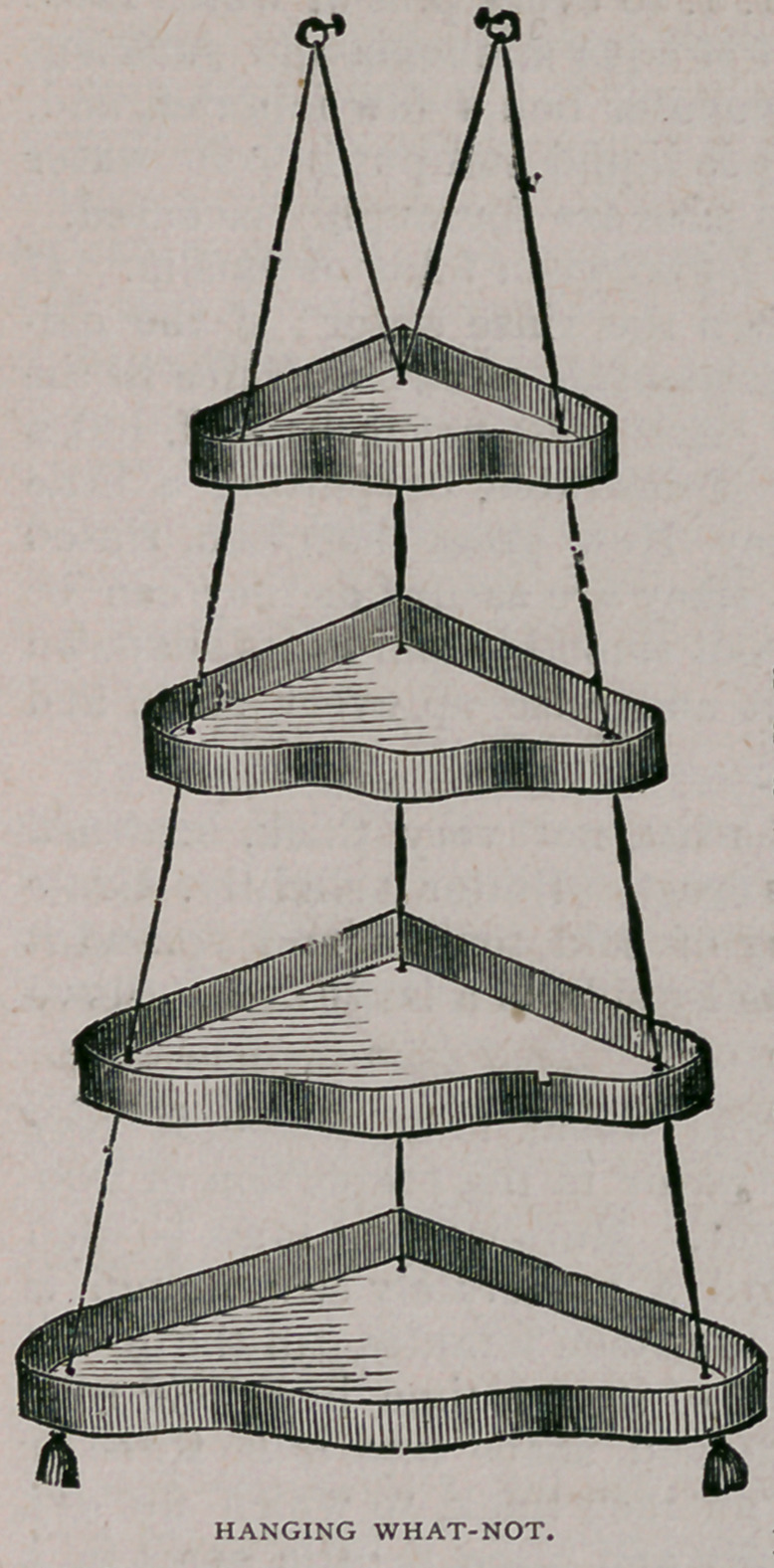# Household

**Published:** 1887-09

**Authors:** 


### HOUSEHOLD.

HANGING WHAT-NOT.—If you want something neat, pretty, useful and very ornamental for your sitting-room, make a corner what-not, like the one shown in our illustration on this page. It can be easily made by anyone, as follows : Get some neat small-figured wall paper, and some nice bordering (eight-strip velvet border is the best), and six or eight yards of the very heaviest picture cord to hang it with. Make the shelves of ½ or ¾ inch boards, planed on both sides, and cut in the form shown or any fancy shape. About sixteen inches wide from the back corner to the front edge (through the middle of the shelf) is the proper width for the lower shelf, and each shelf above should be an inch or an inch and a half narrower than the one next below it. Put a strip on each shelf of thin stuff, as high as the bordering will cover, very stout pasteboard will do. Bore holes in each corner just large enough to admit the cord.

HANGING WHAT-NOT.

In papering be very careful not to leave any blistered places, as they will cause the paper to break. They should be pricked and rubbed down, and the papered surface will stand as much wear as a varnished one. Put bordering on the back and around the front edge, and it is ready to put together. Adjust the cord to the top shelf first, tying a knot under each corner to hold it up ; then hang it up and put on the other shelves in their order, tying a knot under each one. About ten inches or one foot apart is as near together as they should be placed.

If preferred, galvanized wire may be substituted for cord, so bending it at proper intervals as to prevent the sections from slipping downward and packing together.

The French mode of killing poultry, causing instant death and perfect bleeding, is accomplished by opening the beak of the fowl, and with a sharp pointed and narrow bladed knife, making an incision at the back of the roof of the mouth, which will divide the vertebræ and cause immediate death, after which the fowls are hung up by the legs until bled. They are then picked when warm. In this way the skin presents a natural appearance.

Doing Up Lace.—Laces rubbed, starched and ironed are rarely fit to wear again; but good lace may be done up so as to be kept looking fresh long after it is really old and worn.

Laces that require doing up should be basted carefully between folds of thin muslin and put into cold, soft water, to every pint of which must be previously administered a teaspoonful of aqua ammonia and sufficient white soap to make good suds. Let the water boil a few minutes, and, if the laces are not then clean, pour off the liquid and put in cold water as before; continue to do so until the articles are thoroughly cleansed.

Never wring out lace—always squeeze it between folds of muslin. If clear lace is required, put a little bluing in the rinse water; if the old-time yellowish tinge is wished, a few teaspoonfuls of strong coffee in the rinse water will give the requisite hue. Lace must never be stiff, but a little of the limpness may be taken off, if desirable, by putting a little dissolved gum arabic in the rinse water. Now press the clean, rinsed laces between folds of white muslin, till they are as dry as they can be made in this way; then pin each article out smoothly and in its shape on a pillow, and with a fine needle pick out and raise up every stem, and leaf, and thread to its proper place.

Tongue Toast.—Make some slices of toast, not very thick, browned evenly all over on both sides, and minus crust. Butter it slightly. Grate with a large grater a liberal sufficiency of cold tongue and spread it thickly over the toast. Lay the slices side by side in a large dish. Serve at breakfast, luncheon or supper.

Graham Rolls.—Mix graham or whole wheat flour, which, if very coarse, must first be sifted, with ice-cold water in the proportion of two-thirds of a pint of water to a quart of flour. More wetting must be used if the flour is very coarse. Stir fast until a moderately stiff dough is formed, and knead thoroughly from ten to fifteen minutes, till the dough is fine and elastic to the touch. Roll half of it at a time into long rolls a little over an inch in diameter; cut off and shape into rolls three or four inches long and three-quarters of an inch thick, to which no dry flour is left attached. Make them rapidly and place a little apart in a pan; prick them with a fork and put the pan in a hot oven. When done they should not yield to pressure between thumb and finger. They are to be eaten warm or cold, and are just as good rewarmed as when new. To do this dip in cold water, cover with cloth and set in a moderate oven, when they will puff up lighter than at first. These require slow mastication, and are as sweet as a nut and very nutritious.

Snowballs.—Half cup of rice and the same of pearl tapioca, half cup of sugar, a quart of milk, a half teaspoonful of salt, soak rice and tapioca, well mixed together while dry in three cups of water, four hours; salt the milk, dropping in a tiny bit of soda, pour upon the soaked cereals and let them stand together half an hour; set over the fire in a farina kettle and simmer slowly one hour; fill small cups with the mixture while hot, and when cold put on the ice. Turn out in saucers and eat with cream.

## Figures and Tables

**Figure f1:**